# Learning the difficult tasks of caring for dying children

**DOI:** 10.1007/s40037-015-0155-2

**Published:** 2015-02-03

**Authors:** Janet Lefroy

**Affiliations:** Keele University School of Medicine, Stoke-on-Trent, Staffordshire UK

The paper by Arzuaga and Caldarelli [1] in this issue illustrates the principle that in at least some aspects of medical education, being thrown in at the deep end is not the best way to learn to swim. They found that paediatricians can learn difficult things—in this study, to care for dying children—by experience, but for the most difficult aspects a lot of experiences are needed. The risk is that patients and their families can be harmed during this learning process. If families may be left scarred by the trainee’s mistakes, however much the trainee may learn from the experience, ‘One Chance to Get It Right’ [2] applies.

It is therefore reassuring to find that prior education in end-of-life care doubled the comfort levels of trainee paediatricians with a set of difficult end-of-life tasks. Arzuaga and Caldarelli provide evidence that comfort levels correlate with competence. The conclusion is that education in end-of-life care accelerates the acquisition of competence; therefore, we need to ensure that we minimize harm by providing appropriate education.

But comfort is an incongruous term in the context of tragedy and perhaps doctors never (and should never) become comfortable with some situations. Do we expect doctors helping a dying child plus family to ever say that they felt comfortable with these tasks? One can think of reasons for increased comfort as the years go by—discussions/debriefings helping to enhance coping mechanisms; watching role models; experience being the teacher and experience also inuring the doctor to the pain. Might it also be that senior doctors reading the questionnaire feel they ‘ought’ to be comfortable because they are senior? The word comfortable may have the wrong resonance for others. Comfortable is not the same as confident or competent. In fact, a doctor who feels comfortable may appear to the desperate family to be nonchalant and not communicating well. The family do not want the doctor to be comfortable. They want those caring for them and their child to be caring and competent. This is clearly the intention of the authors of this study. One is reminded that it is always wise to pilot a questionnaire with a few members of the target participant population to ensure that their understanding of the terminology accords with the researchers’ intentions.

Before addressing what appropriate end-of-life care training might look like, it is worth taking a closer look at what this study found to be the impact of experience and the impact of education.

Experience helped to improve comfort in some duties of paediatricians caring for the dying child, but they remained uncomfortable with others until some years later in their career. A pattern emerges. The times when experience failed to make paediatricians more comfortable were when there was the potential of conflict with their young patient’s family. These tasks included discussing options. One can imagine that a difficult experience involving conflict might only serve to highlight deficiencies and make a doctor feel less comfortable. Comfort did, however, increase with experience in the technical skills of prescribing at the end of life and confirming death.

Likewise, prior education did improve comfort for this set of doctors in all except two tasks. One was managing the difficult family and here not even the most experienced paediatricians felt comfortable. The other was confirming death—a task with which paediatricians did become comfortable but which was learned by experience. Whether their prior education had addressed these specific aspects of end-of-life care is unknown.

Doctors’ self-assessed educational needs in this study confirmed that they needed to learn the skills of communicating with the family. What sort of education is likely to help future paediatricians (and all doctors) to be able to handle conflict in discussions over patient management? If someone told me they felt uncomfortable, I would ask about the source of that discomfort. Did it stem from emotion and require debriefing after the event? Did it stem from lack of skills and require role models plus observed practice and feedback to improve skills? Or did it stem from uncertainty about facts such as the law or about the ethics of a scenario and require discussion/clarification?

Using trainees’ experiences as the substrate for learning is often very helpful in providing learning which is directly relevant to the perceived need of the individual with the story and also credible to other learners because of the authentic context. Doctors can be encouraged to write personal accounts of trying to do these tasks and their reflections on how they might have done better. They can also consider role modelling they have witnessed of how these were handled well or badly by others. This is what Schon terms ‘reflection-on-action’ [3].

Reflection-on-action, however, requires experience—‘actions’ on which to reflect. How can we safely provide that experience for learners in the high-stakes situation of communicating with families of dying children—a situation in which there is ‘one chance to get it right’? Education can ensure a baseline competence before learners approach the task in a real setting. In such a situation the medical educator should consider simulation for a safe environment in which to experiment, get feedback and practice until satisfied that it would be well done in real life. This is likely to need small-group learning for safe discussion, role play and feedback. Starting with lesser challenges building to greater and allowing the role play to stop for discussion, advice, rewinding and attempting different approaches can help a great deal. Learners can reflect on the simulated experience without regret, note their improving competence and thus build self-efficacy levels.

More specifically, simulation (role play) can be used to learn skills for discussing options and handling conflict using communication strategies such as negotiation. There are several suitable ‘toolkits’ available for negotiation and shared decision-making [4, 5]. The skills of negotiation involve understanding the viewpoint of each party and working from common ground to explore available options and likely outcomes in order to reach a shared decision.

Most senior doctors did not learn this way. But learning by trial and error with patients and their families is not acceptable at the end of life. It is imperative that we educate our successors better than we ourselves were educated. Once they have learned a baseline level competence, a commitment to ongoing educational strategies such as reflection-on-action and debriefing with colleagues will then be important in their continued learning, refining, and improving of skills during their training and throughout their professional career.
